# The Need to Handicap the Recipient's Native Liver in the Rat Model of Heterotopic Auxiliary Liver Transplantation

**DOI:** 10.1155/1999/12802

**Published:** 1999-07

**Authors:** Ye-Dong Fan, Marleen Praet, Bernard De Hemptinne

**Affiliations:** 1 Department of Surgery Division of Experimental Surgery Belgium; 2 Department of Pathology University Hospital of Ghent Belgium; 3 Department of Surgery Division of Experimental Surgery University Hospital of Ghent De Pintelaan 185 Ghent B-9000 Belgium

## Abstract

In the rat model of heterotopic auxiliary liver transplantation (HALTx), the opinion varies on whether and how the recipient's native liver should be handicapped. To avoid atrophy of the transplanted organ, in this study, two different handicaps were evaluated and their effects on post-operative animal survival and liver biology are described. With a sole portacaval shunt (group 1) all rats survived longer than 3 months. An additional handicap of the liver with either a 68% partial hepatectomy (68% PH) (group 2), or both a 68% PH and a common bile duct ligation (CBDL) (group 3) led to a 100% mortality within 2 days after surgery. When an auxiliary liver was transplanted to the rats handicapped with a 68% PH (group 4), serum Bilirubin and ALAT values were significantly lower than those handicapped with both a 68% PH and a CBDL (group 5). Autopsy and histology of the long-term survivors revealed the atrophy of the engrafted livers and the regeneration of the native livers in group 4, whereas it showed the opposite in group 5. Thus the various manipulations of the native liver do influence differently the post-transplant animal survival, serum liver biochemistry and the outcome of the engrafted liver in this rat model of HALTx.


*HPB Surgery*, 1999, Vol. 11, pp. 225–234
Reprints available directly from the publisher
Photocopying permitted by license only

© 1999 OPA (Overseas Publishers Association) N.V.
Published by license under
the Harwood Academic Publishers imprint,
part of The Gordon and Breach Publishing Group.
Printed in Malaysia.

### The Need to Handicap the Recipient's Native Liver in the Rat Model of Heterotopic Auxiliary Liver Transplantation

YE-DONG FAN[a,*], MARLEEN PRAET[b] and BERNARD DE HEMPTINNE[a]

[a] *Department of Surgery, Division of Experimental Surgery;* [b] *Department of Pathology, University Hospital of Ghent, Belgium*

*(Received 2 November 1997; In final form 20 March 1998)*

**In the rat model of heterotopic auxiliary liver transplantation (HALTx), the opinion varies on whether and how the recipient's native liver should be handicapped. To avoid atrophy of the transplanted organ, in this study, two different handicaps were evaluated and their effects on post-operative animal survival and liver biology are described. With a sole portacaval shunt (group 1) all rats survived longer than 3 months. An additional handicap of the liver with either a 68% partial hepatectomy (68% PH) (group 2), or both a 68% PH and a common bile duct ligation (CBDL) (group 3) led to a 100% mortality within 2 days after surgery. When an auxiliary liver was transplanted to the rats handicapped with a 68% PH (group 4), serum Bilirubin and ALAT values were significantly lower than those handicapped with both a 68% PH and a CBDL (group 5). Autopsy and histology of the long-term survivors revealed the atrophy of the engrafted livers and the regeneration of the native livers in group 4, whereas it showed the opposite in group 5. Thus the various manipulations of the native liver do influence differently the post-transplant animal survival, serum liver biochemistry and the outcome of the engrafted liver in this rat model of HALTx.**

*Keywords:* Heterotopic auxiliary liver transplantation, handicap of the native liver, portacaval shunt, partial hepatectomy, common bile duct ligation, graft atrophy, inter-liver competition

### INTRODUCTION

In recent years, more and more reports from different organ-transplantation centers have been published with encouraging results, using heterotopic auxiliary liver transplantation (HALTx) to treat patients with end-stage liver diseases especially fulminant and subfulminant hepatic failure [1–3]. This has raised a new interest to study the potential problems of this operative procedure with animal models [4, 5]. HALTx in the rat has long been used for experimental research, since it was first described by Lee and Edgington in 1966 [6]. The investigators have used this model to study amongst others, liver regeneration, hepatotrophic factors, allogenic microchimerism and graft rejection [5,7,8]. Several technique modifications of this model have been introduced [9–12].

*Address for correspondence: Department of Surgery, Division of Experimental Surgery, University Hospital of Ghent, De Pintelaan 185, B-9000 Ghent, Belgium.

Although the observation of graft atrophy and the concept of "inter-liver competition" in HALTx were reported in the early 60's [13–15], opinion still varies on whether in this rat model the native liver of the recipient should be handicapped and in this case which approach should be used. Some authors handicapped the native liver either with a 68% partial hepatectomy (68% PH) [7, 10] or a 68% PH plus a common bile duct ligation (CBDL) [9, 11]. Others, on the contrary, left the native liver intact [5, 8, 12]. Up to date, there has been no report about how these different manipulations of the native liver do influence the post-HALTx animal survival and serum biochemical values. This was investigated in the present study.

### MATERIALS AND METHODS

### Experimental Design (Table)

To evaluate the effects of different handicaps on the native liver, three experimental groups were studied, in which the livers were put in functionally similar situations as the native livers in the HALTx model: group 1 ($n = 10$), an end-to-side portacaval shunt (PCS) (without further handicap of the liver); group 2 ($n = 10$), a PCS plus a 68% PH as additional handicap of the liver; and group 3 ($n = 10$), a PCS, a 68% PH and a CBDL to handicap the liver.

Two experimental groups were tested later on, since no long-term survivor and thus serum biochemical evolution could be obtained from the rats of groups 2 and 3. In group 4 ($n = 12$), a HALTx was performed after the native liver was handicapped in the same way as in group 2 (PCS and 68% PH). In group 5 ($n = 12$), HALTx was performed after the handicap of the native liver as in group 3 (PCS, 68% PH and CBDL).

### Experimental Animals

Male Wistar rats weighing 250–340 g were purchased from Janssen Pharmaceutica N.V., Belgium. Donor and recipient rats were matched for size. Rats had free access to food and water prior to surgery. All animals received humane care as outlined in the *Guide for the Care and Use of Laboratory Animals* (NIH Publication No. 86–23, revised 1985).

### Surgical Techniques

All operations were carried out under ether anaesthesia with clean but non sterile instruments and x 12.5 magnification.

(a) 68% PH: The median lobe and the left lobe of the liver were ligated and then excised according to the standard method introduced by Higgins and Anderson [16].
(b) CBDL: A segment of the common bile duct was removed after double ligation.
(c) PCS: The standard method described by Lee [17] was modified using the cuff technique to perform a PCS. After a left nephrectomy, the left renal vein was freed up to the vena cava which was then cross clamped. An incision was made into the anterior wall of the stump of the left renal vein. The portal vein was divided from the pyloric vein to the liver hilus, tied, transsected and cuffed with a 0.4 cm length polyethylene tube. The cuffed portal vein was then inserted into the left renal vein and secured with a 5–0 ligature.
(d) HALTx: The standard technique described by Marni [9] was used with a slight modification. After the donor liver was perfused with heparinized 4°C physiological saline solution via the portal vein, 30% of the liver was used for transplantation. The native liver of the recipient rat was handicapped with a 68% PH or both a 68% PH and a CBDL according to the study group, while the hepatic artery remained intact. The engrafted liver was then implanted in the right paravertebral gutter under the native liver of the recipient. The cuff technique described by Kamada [18] was used for the anastomoses of both the vena cava and the portal vein. The engrafted liver received its blood supply from the portal vein of the recipient. The venous drainage was made through the right renal vein of the recipient to the vena cava. Restoration of the bile drainage was done by insertion of the intubated common bile duct of the donor liver into the recipient duodenum. No re-arterialization was performed. To this standard procedure, we added a fixation of the remaining donor diaphragm to the recipient lateral abdominal wall in order to avoid twisting and kinking of the donor vena cava.

The duration of the different operative phases (mainly the portal vein and the vena cava cross-clamping time) was comparable among groups 1, 2 and 3. The cold preservation time of the grafts (starting from the saline perfusion via the portal vein ending at the portal re-vascularization of the engrafted liver) was kept between 72 and 79 minutes in the rats of HALTx groups 4 and 5.

### Post-operative Care

All rats were given 10 mg Na Cefazolin intramuscularly after the operation and this was repeated daily for three days. No immunosuppressive agent was administered to the rats. During the following twenty-four hours, rats were allowed to drink 5% glucose, after which the pre-operative diet of food pellets and water was resumed.

### Follow-up

Body Weight was recorded daily and survival time was determined.

For the biochemical analysis, a blood sample of 0.4–0.5 ml was collected from the tail vein. Serum total bilirubin (TBil) and alanine aminotransferase (ALAT) were determined pre-operatively, on post-operative day 3, weekly for one month and monthly for 3 months.

An autopsy was performed whenever a rat died to identify possible complications. The livers were removed at sacrifice and fixed with 10% formalin. Four micron sections were stained with hematoxylin and eosin (HE) or trichrome. Liver samples were studied by light microscopy.

### Statistical Analysis

The data were expressed as the mean $\pm$ standard deviation (SD). The Student's $t$-test was used for the data analysis. $P$ values equal to or less than 0.05 were considered statistically significant.

### RESULTS

### Body Weight and Animal Survival

The body weight of all rats declined 24 hours after the operation with average $10.00 \pm 6.47$ grams per rat in group 4 and $18.33 \pm 14.03$ grams in group 5 (NS). Three weeks later, the rats of group 4 regained their pre-operative weight and even exceeded up to average $24.07 \pm 3.40$ grams compared with $1.67 \pm 11.16$ grams in group 5 (NS).

As shown in the Table, the rats of group 1 could well tolerate a PCS whereas all rats in groups 2 and 3 died of liver failure within two days after the surgery.

In group 4, the 1-month survival rate was as high as 92%. Only two rats died of abscess in the native liver or in both the native and the engrafted livers on the 17th and 34th post-operative day. In group 5, only 50% of the animal survived longer than 3 months. Except a rat that died of rejection on day 23, all others died of infectious complications (cholestasis in 1, abscess in 3 and peritonitis in 1).

### Serum Biochemical Parameters

Serum TBil of the rats in group 5 was significantly higher than those of group 4 on post-

TABLE Experimental groups and post-operative survival

| Experimental groups* | Survival time | | | | |
|---|---|---|---|---|---|
| G1 ($n=10$): PCS | | | >3 months (10/10) | | |
| G2 ($n=10$): PCS + 68% PH | | | 0.81 ± 0.56 days (*mean* ± *SD*) | | |
| G3 ($n=10$): PCS + 68% PH + CBDL | | | 0.51 ± 0.30 days (*mean* ± *SD*) | | |
| | | | *Survival rates* | | |
| | 1 *week* | 2 *weeks* | 3 *weeks* | 1 *month* | 2 *months* |
| G4 ($n=12$): HALTx + 68% PH | 100% | 100% | 92% | 92% | 83% |
| G5 ($n=12$): HALTx + 68% PH + CBDL | 100% | 83% | 58% | 50% | 50% |

* PCS: portacaval shunt; 68% PH: 68% partial hepatectomy; CBDL: common bile duct ligation; HALTx: heterotopic auxiliary liver transplantation.

operative day 7 ($p=0.025$) and 28 ($p=0.02$) (Fig. 1a). The rats ($2^{\#}, 3^{\#}, 8^{\#}$ and $10^{\#}$) of group 5, that had the highest values of TBil on day 7, developed infectious complications and died within the following weeks. By disregarding the values of those rats that died of complications, the differences between the two groups were still recorded but they were no longer statistically significant (Fig. 1b). The high TBil peak of group 5 on day 28 was this time due to the two rats that developed secondary biliary cirrhosis of the engrafted livers found later at autopsy.

As showed in Figure 2a, significant differences of mean serum ALAT between the two groups were found on day 3 ($P=0.014$), 21

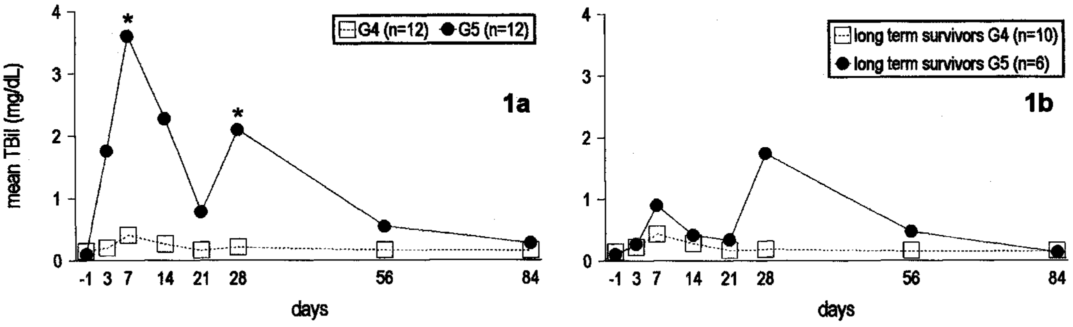

FIGURE 1 Comparison of the mean serum total bilirubin (TBil) between groups 4 and 5 post-HALTx: (a) Data of the whole group. Statistically significant differences were found on day 7 ($p=0.025$) and day 28 ($p=0.02$); (b) Data of the long term survivors. No significant differences between the two groups could be seen.

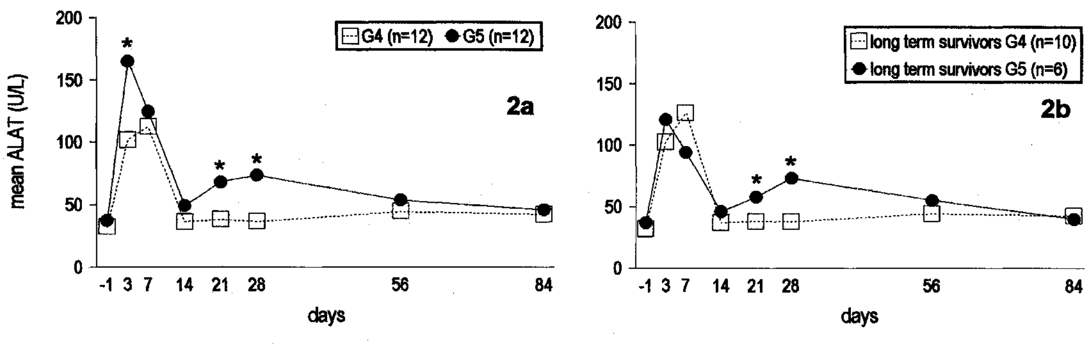

FIGURE 2 Comparison of the mean serum alanine aminotransferase (ALAT) between groups 4 and 5 post-HALTx: (a) Data of the whole group. The statistically significant differences were found on day 3 ($p=0.014$), day 21 ($p=0.014$) and day 28 ($p<0.001$); (b) Data of the long term survivors. The differences between the two groups was not significant on day 3 and remained significant on day 21 ($p=0.008$) and 28 ($p<0.001$).

( $=0.014$) and 28 ($p<0.001$). Similarly to serum TBil, the highest ALAT values of group 5 on day 3 were again due to the rats that later died of infectious complications. When those values were excluded, the differences were no longer significant on day 3 but remained significant on day 21 ($p=0.008$) and 28 ($p<0.001$).

### Autopsy and Histology

At the end of three months observation period, all the engrafted livers of the 10 survivors in group 4 showed atrophy at sacrifice while their native livers showed normal appearance (Fig. 3a). The general architecture of those engrafted livers was altered by fibrosis and ductular metaplasia. Only a few groups of hepatocytes were present (Fig. 4a).

On the contrary, in group 5, the engrafted livers of all but 2 long term survivors appeared normal macroscopically whereas their native livers showed clear atrophy (Fig. 3b). The general architecture of those engrafted livers was well preserved without major histological alterations (Fig. 4b). The 2 remaining rats ($1^{\#}$ and $6^{\#}$) presented cirrhotic appearance of their engrafted livers and the histology revealed secondary biliary cirrhosis due to sub-obstruction of their hepaticoduodenostomy.

### DISCUSSION

In the previous studies of rat HALTx, the postoperative animal survival and serum biochemistry were most commonly analysed without taking into account the role of the handicap of the native liver. In this study, we show for the first time how these handicaps can differently influence the post-transplant biochemical parameters.

The classical rat model of HALTx includes a complete shunting of the portal blood away from the native liver to re-vascularize the engrafted liver. In this case, the blood supply of

(a)

(b)

FIGURE 3 Autopsy. (a) a rat of group 4 (HALTx with a 68% PH to handicap the native liver) sacrificed 7 months after the operation: the engrafted liver (↗) atrophied whereas the native liver had normal appearance; (b) a rat of group 5 (HALTx with a 68% PH and a CBDL to handicap the native liver) sacrificed 12 months after the operation: the size of the engrafted liver (↗) enlarged and the native liver underwent complete atrophy. (See Color Plate I).

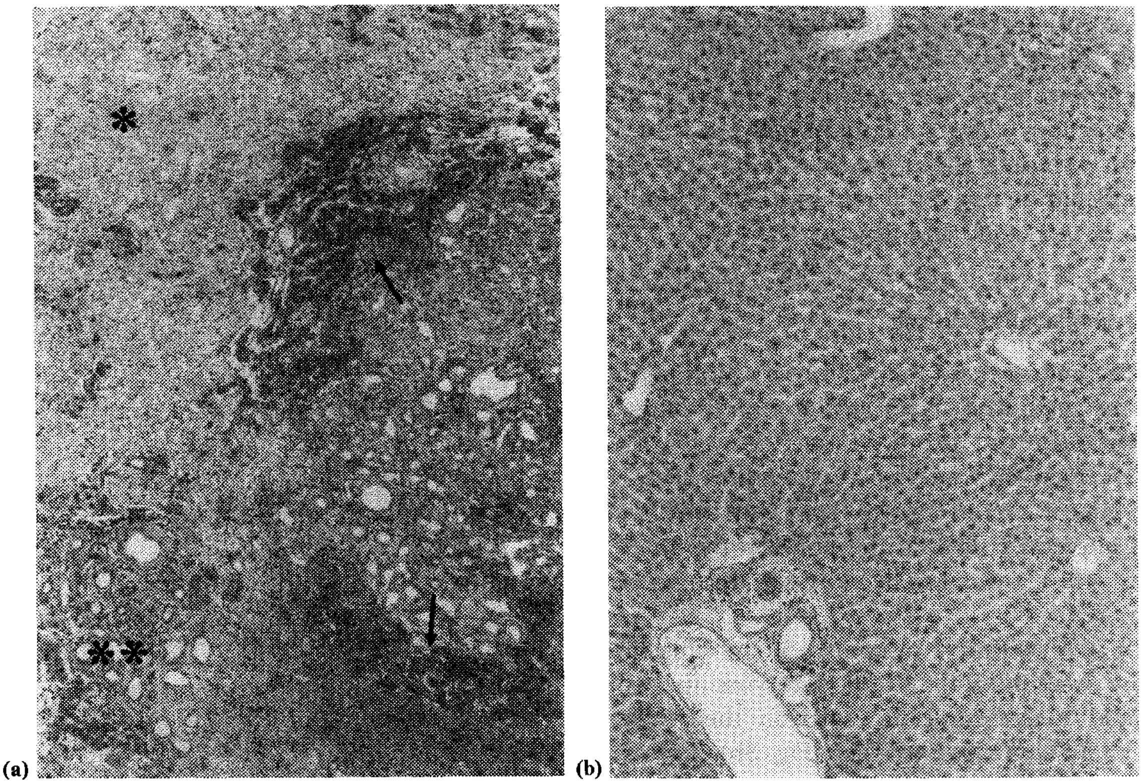

FIGURE 4 Histological observations. (a) a rat of group 4 (HALTx with a 68% PH to handicap the native liver) sacrificed 7 months after the operation: the general architecture of the engrafted liver was altered by fibrosis (*) and ductular metaplasia (**). Only a few groups of hepatocytes were present (↗) (Trichrome, x 10); (b) a rat of group 5 (HALTx with a 68% PH and a CBDL to handicap the native liver) sacrificed 12 months after the operation: the general architecture of the engrafted liver was well preserved and no major alterations were present (HE, x 10).

the native liver is solely given by the hepatic artery, which corresponds effectively to a PCS of the liver. It appears that the blood flow of the hepatic artery can compensate the by-passed portal blood after a PCS and maintain a "functioning liver", so that the effects of PCS do not significantly influence the animal survival. Without handicap of the native liver, the engrafted liver is therefore transplanted parallel with a normal functioning liver. The increased hepatic blood flow will not be sufficient to support proper hepatic function if a further handicap is carried out in the native liver. It is reported that in the case of a 68% PH added to an end-to-side PCS, the rat survival does not exceed 2 days [11]. The results of the present study confirm this data and show, as would have been anticipated, that the combination of a PCS, a 68% PH and a CBDL does even shorten this survival time. In both these circumstances, no animal can be kept alive unless an auxiliary liver graft is transplanted.

The long-term survivors in the HALTx groups make it possible to perform a comparative study of the serum biochemistry between the rats bearing different liver handicaps. When the rats were handicapped only with a 68% PH as in group 4, ALAT increased to a peak level 1 week post-transplant but then returned to normal values after 2 weeks reflecting transitory hepatocellular damage. Both ischemic injury of the graft caused by preservation and the surgical manipulations of the liver at the time of 68% PH are responsible for this initial elevation. On the other hand, TBil of this group, only raised slightly after the surgery even in the rats that presented fatal complications. This demonstrates that in case of a sole 68% PH to handicap the native liver, either one of the two livers can still compensate for the functional deficiency of the other. An additional CBDL to the native liver will handicap this organ so profoundly that it can no longer compensate when the engrafted liver is affected by an acute infectious complication: TBil sharply increased in these cases followed by the animal deaths shortly later as shown in group 5. The "double handicap" of the native liver is also responsible for a second peak of TBil around 1 month after HALTx. The rats that developed secondary biliary cirrhosis of the graft presented the highest values at this time point, when the exhaustion of the regeneration potential and thereby the functional failure of the native liver occurred [19]. The repermeabilisation of the extrahepatic biliary duct of the native liver may presumably explain the normalisation of serum TBil in the long-term survivors.

It has been found that long-standing bile duct obstruction of the native liver impairs the function of reticuloendothelial system which might lead to serious post-operative complications [20, 21]. The rats handicapped with CBDL (group 5) had indeed a higher incidence of infection and more pronounced post-operative weight-loss. Cholestasis induced by CBDL also inhibits the metabolic functions of liver cells including mitochondrial function. As consequence, regeneration capacity of this organ is compromised [22,23]. These may be the reasons why a CBDL together with a 68% PH can affect the native liver to such a degree that the post-HALTx animal survival and normal serum biochemical values depend totally upon the functionality of the engrafted liver.

HALTx has advantages over OLTx in treating acute liver failure, inborn errors of metabolism and, exceptionally, chronic liver diseases with high operative risk for OLTx. In the case of acute liver failure, the diseased native liver is expected to recover while the engrafted liver is providing functional support. The engrafted liver will then be removed or left in place to atrophy if the native liver resumes its proper function [1–3]. For patients with chronic liver diseases, regeneration of the engrafted liver and atrophy of the native liver is the rule after a HALTx [24]. Although up to now no clinical experience with HALTx to treat patients with inborn errors of metabolism has been reported, the long term functionality of both the engrafted and the native livers should be looked for [4]. In view of these different situations, it is indeed important to have a better understanding about the mechanisms of inter-liver competition and the right approaches to control or to modify this process. It is for this purpose, the rat HALTx can be an interesting model to study the insight of this phenomenon, even though it does not replicate the exact clinical situation. It is generally accepted that proper regeneration of the liver can only be achieved when the portal vein is re-vascularized with venous blood or through arterialization of the portal stump [6,15,25]. Interestingly, the partially resected native liver can still regain progressively its volume and function despite the lack of portal blood flow, whereas the engrafted liver eventually atrophies. In case of an additional handicap of the native liver, by means of a CBDL, the functional recuperation of the native liver is completely impaired while the engrafted liver takes over the function and keeps its normal macroscopic and microscopic aspects. These results suggest that besides the known hepatotrophic factors [8,26], the diseased state of the native liver also influences the outcome of the inter-liver competition. Yu and co-workers have shown that the atrophy of the engrafted liver can be prevented by its re-arterialisation even when the native liver is not handicapped [5]. Re-arterialisation of the graft in the OLTx model reduces biliary complications, modifies the immunological response and improves micro-vascular perfusion of the graft [27–29]. In the rat model of HALTx, these beneficial effects certainly favour the competitive position of the engrafted liver.

The data of the present study show that in the rat model of HALTx the different manipulations on the native liver do influence not only the post-transplant animal survival but also the serum biochemical values of the liver. Furthermore, handicap of the native liver limited to a 68% PH will lead to the atrophy of the engrafted liver, whereas a 68% PH combined with a CBDL is an handicap that impairs the regeneration of the native liver. In the latter case, the post-HALTx survival and normalisation of the serum biochemistry depend solely upon the viability of the engrafted liver. Although this model has no direct similarity with clinical condition, it allows further approach and understanding to the concept of inter-liver competition.

### *References*

[1] Moritz, M. J., Jarred, B. E., Armenia, V., Radomski, J., Carabasi, R. A., Zeitoun, G., Columbus, K., Rubin, R. and Smaddrey, W. (1990). Heterotopic liver transplantation for fulminant hepatic failure-a bridge to recovery. *Transplantation*, **50–3**, 524–526.

[2] Van Hoek, B., Ringers, J., Kroes, A. C., van Krieken, J. H., van Schelven, W. D., Masclee, Ad., van Krikken-Hogenberk, L. G., Haak, H. R., Lamers, C. B. and Terpstra, O. T. (1995). Temporary heterotopic auxiliary liver transplantation for fulminant hepatitis B. *Journal of Hepatology*, **23**, 109–118.

[3] Boudjema, K., Cherqui, D., Jaeck, D., Chenard-Neu, M. P., Steib, A., Freis, G., Becmeur, F., Brunot, B., Simeoni, U., Bellocq, J. P., Tempe, J. D., Wolf, P. and Cinqualbre, J. (1995). Auxiliary liver transplantation for fulminant and subfulminant hepatic failure. *Transplantation*, **59–2**, 218–223.

[4] Madern, G. C., Terpstra, O. T., Sinaasappel, M., Provoost, A. P., Rothuizen, J. and Molenaar, J. C. (1991). Heterotopic liver transplantation corrects the inborn error of hepatic metabolism in a dog model. *Transplantation Proceedings*, **23–1**, 716–717.

[5] Yu, W. Y., Wan, X. Y., Wright, J. R., Coddington, D. and Bitter-Suermann, H. (1994). Heterotopic liver transplantation in rats: Effects of intrahepatic islet isografts and split portal blood flow on liver integrity after auxiliary liver isotransplantation. *Surgery*, **115–1**, 108–117.

[6] Lee, S. and Edgington, T. S. (1966). Liver transplantation in the rat. *Surgical Forum*, **17**, 220–222.

[7] Lee, S. and Edgington, T. S. (1968). Heterotopic liver transplantation utilising inbred rat strains: I. Characterisation of allogeneic graft rejection and the effects of biliary obstruction and portal vein circulation on liver regeneration. *American Journal of Pathology*, **52–3**, 649–669.

[8] Bestian, J. M., Janin, A., Zenner, L., Pruvot, F. R., Zelus, D., Courtade, A., Dessaint, J. P. and Capron, A. (1995). Allogenic microchimerism following auxiliary heterotopic liver transplantation in rat and swine. *Transplantation Proceedings*, **27–2**, 1675.

[9] Marni, A. and Ferrero, M. E. (1985). Heterotopic liver grafting in the rat: A simplified method using cuff techniques. *Transplantation*, **39–3**, 329–331.

[10] Kort, W. J., Wolff, E. D. and Eastham, W. N. (1971). Heterotopic auxiliary liver transplantation in rats. *Transplantation*, **12–6**, 415–420.

[11] Hess, F., Jerusalem, C. and van der Heyde, M. N. (1972). Advantages of auxiliary liver homo-transplantation in rats. *Archives Surgery*, **104**, 76–80.

[12] Müller, G. (1983). A simple technique for heterotopic auxiliary liver transplantation in the rat. *Transplantation*, **36–2**, 221–222.

[13] Starzl, T. E., Marchioso, T. L. and Huntley, R. T. (1964). Experimental and clinical homo-transplantation of the liver. *American New York Academic Science*, **120**, 739–765.

[14] Marchioro, T. Z., Poster, K. A., Dickinson, T. C., Faris, T. D. and Starzl, T. E. (1965). Physiologic requirements for auxiliary liver transplantation. *Surgery, Gynecology and Obstetrics*, **121**, 17–31.

[15] Van der Heyde, M. N., Schalm, L. and Vink, M. (1967). The role of functional competition in auxiliary liver transplantation. *Transplantation*, **5**, 78–80.

[16] Higgins, G. M. and Anderson, R. M. (1931). Experimental pathology of the liver: I. Restoration of the liver of the white rat following partial surgical removal. *Archives Pathology*, **12**, 186–202.

[17] Lee, S. and Fisher, B., Portacaval shunt in the rat. *Surgery* 1961; 50:668–672.

[18] Kamada, N. and Calne, R. Y. (1983). A surgical experience with five hundred thirty liver transplantation in the rat. *Surgery*, **93–1**, 64–69.

[19] Gross, J. B., Reichen, J., Zeltner, T. and Zimmermann, A. (1987). The evolution of changes in quantitative liver function tests in a rat model of cirrhosis: correlation with morphometric measurement of hepatocyte mass. *Hepatology*, **7**, 457–63.

[20] Holman, J. M. Jr. and Rikkers, L. F. (1982). Biliary obstruction and host defence failure. *Journal of Surgical Research*, **32**, 208–213.

[21] Andersson, R. and Foss, A. (1991). Abdominal sepsis following liver resection in the rat. *Hepatogastroenterology*, **6**, 547–549.

[22] Koyaman, K., Takagi, Y., Ito, K. and Sato, T. (1981). Experimental and clinical studies on the effect of biliary drainage in obstructive jaundice. *American Journal of Surgery*, **142**, 293–299.

[23] Miyata, K. (1983). Delayed recovery of mitochondrial function in rat liver after releasing biliary obstruction. *Nagoya Journal of Medical Science*, **45**, 97–105.

[24] Terpstra, O. T., Schalm, S. W., Weimar, W., Willemse, P., Baumgartner, D., Groenland, T., ten Kate, F., Porte, R. J., De Rave, S., Reuvers, C. B., Stibbe, J. and Terpstra, J. L. (1988). Auxiliary partial liver transplantation for end-stage chronic liver disease. *New England Journal of Medicine*, **319**, 1507–1511.
[25] de Hemptinne, B. (1980). Acute effects of portacaval shunt and arterialization on rat hepatic regeneration. *European Surgical Research*, **12** (Suppl 1), 3.
[26] Terpstra, O. T., Reuvers, C. B. and Schalm, S. W. (1988). Auxiliary heterotopic liver transplantation. *Transplantation*, **45**, 1003–1007.
[27] Howden, B., Jablonski, P., Grossman, H. and Marsshall, V. C. (1989). The importance of the hepatic artery in rat liver transplantation. *Transplantation*, **47**, 428–431.
[28] Engemann, R., Ulbrichs, K., Thiede, A., Müller-Ruchholtz, W. and Hamelmann, H. (1982). Value of a physiological liver transplant model in rats. *Transplantation*, **33**, 566–568.
[29] Post, S., Menger, M. D., Rentsch, M., Gonzalez, A. P., Herfarth, C. and Messmer, K. (1992). The impact of arterialization on hepatic microcirculation and leukocyte accumulation after liver transplantation in the rat. *Transplantation*, **54**, 789–794.

### COMMENTARY

"The necessity to handicap the recipient's native liver in the rat model of heterotopic auxillary liver transplantation".

Auxillary liver transplantation is an attractive concept in certain clinical situations. These include acute fulminant liver failure in which regeneration of the diseased liver potentially allows withdrawal of immunosuppression with or without removal of the graft, as well as in born errors of metabolism without underlying structural liver abnormalities. The potential role in patients with cirrhosis is uncertain because of the very high late incidence of malignant transformation in the native liver. A major problem in auxillary transplantation is the competition between the grafted liver and the host liver which has caused confusion both experimentally and clinically. This paper explores the variation in experimental models of rat auxillary transplantation and has produced convincing experimental data as to how the native liver should be handicapped in order to fully explore the competition between both the native and grafted liver. This paper is a useful contribution to our knowledge on this confusing subject and will help set standards for future rat experimental studies on this topic. It should be remembered however that this work is far removed from the clinical setting and any conclusions reached will not have any direct applications to clinical practice.

Mr. JAC Buckels
University Department of Surgery
University of Birmingham
Queen Elizabeth Hospital
Edgbaston
BIRMINGHAM B12 2TH

In this paper Y.-D. Fan and colleagues investigate several modalities of trauma to the liver of the rat after which the animal receives an auxiliary graft, heterotopically placed. Although their technique of impairment of the native liver is not similar to the clinical situation (as they state in the Discussion) it offers an interesting opportunity to study the physiology of "functional competition" between two livers. Since the first description of this phenomenon in rabbits in which one lobe of the liver hypertrophied when the other lobe was handicapped by bile duct of portal vein branch ligation (L. Schalm *et al.*, Gastroenterology, 1956; 31: 131–155) a debate started on the importance of the role of portal venous blood. In a classical paper Marchioro, Starzel a.o. (Surg Gyn Obst 1965; 121: 17–31) demonstrated the presence of specific nutrients in portal venous blood, called "hepatotrophic factors", necessary to maintain the integrity of the liver and to stimulate compensatory growth after damage to the liver. Although the true nature of the hepatotrophic factors is still not elucidated several hormones, growth factors and cytokines are among them. Fan and coworkers demonstrated that the native liver with a dual blood flow is capable of compensatory hyperplasia after 2/3 hepatectomy unless an extra trauma such as common bile duct ligation is added. Then, the liver graft supplied by portal blood only, will benefit from the hepatotrophic factors while the native liver becomes fibrotic.

It is likely that the upregulation of genes that express the receptors that bind the different growth factors occurs in the former situation in the native liver, while in the more handicapped situation the recipient's own liver is too damaged to up-regulate those gene(s). The growth factors will then bind to the grafted liver and the stimulus to proliferate will be exercised in the graft.

The model as described in the paper enables further study into the physiology of inter-liver competition.

Prof. O. Terpestra
Department of Surgery
University of Leiden
P.O. Box 9600
Rijnsburgerweg 10
LEIDEN 2300 RC
Netherlands

